# Seed-Borne Endophytes and Their Host Effects

**DOI:** 10.3390/microorganisms13040842

**Published:** 2025-04-07

**Authors:** Hongyan Hu, Shucun Geng, Youyong Zhu, Xiahong He, Xiaoxia Pan, Mingzhi Yang

**Affiliations:** 1 School of Ecology and Environmental Science, Yunnan University, Kunming 650504, China; hucarrot1215@163.com (H.H.); gengshucun99@163.com (S.G.); 2 Institute of International Rivers and Ecological Security, Yunnan University, Kunming 650504, China; 3 College of Plant Protection, Yunnan Agricultural University, Kunming 650201, China; yyzhu@ynau.edu.cn; 4 Southwest United Graduate School, Kunming 650092, China; 5 Key Laboratory of Forest Resources Conservation and Utilization in the Southwest Mountains of China Ministry of Education, Southwest Forestry University, Kunming 650224, China; hexiahong@hotmail.com; 6 Key Laboratory of Chemistry in Ethnic Medicinal Resources, State Ethnic Affairs Commission & Ministry of Education, School of Ethnic Medicine, Yunnan Minzu University, Kunming 650504, China

**Keywords:** seed-borne endophytes, diversity, vertical transmission, plant-endophyte interaction, host effect

## Abstract

In the process of long-term co-evolution, endophytes and host plants benefit from and interact with each other, resulting in positive effects such as promoting plant growth, enhancing resistance, producing beneficial secondary metabolites, and negative effects such as carrying pathogens and producing toxins. In addition to the vegetative organs, plant seeds are also colonized by diverse endophytes and serve as vectors for the transmission of endophytes across plant generations. Seed endophytes, termed seed-borne endophytes (SBEs), have attracted much attention because these endophytes are involved in the assembly of the plant association microbiome and exert effects on progeny plants through vertical transfer. However, the importance of SBEs may still be underestimated. The present paper reviews the diversity, origin, and vertical transmission of seed endophytes, as well as their interaction and function with hosts, so as to provide a reference for future research and application of seed endophytes.

## 1. Introduction

Since the mid-19th century, the definition of plant endophyte has been continuously improved and developed, and Orlando Petrini’s definition of endophytes as “all organisms inhabiting plant organs that, at some stage in their life cycle, can colonize internal plant tissues without causing apparent harm to their host” [1], has been widely accepted. However, this definition of endophyte in application has the problem of identifying the pathogenicity of microorganisms inhabiting plant tissues, so researchers have suggested that endophytes should be defined as microorganisms inhabiting plant tissues for all or part of their life history, regardless of their function, including bacteria, actinomycetes, and fungi [2]. Endophytes can be widely found in different organs and tissues of plants such as roots, stems, leaves, flowers, fruits, and seeds, among which seeds are important means of production and reproductive organs, and the endophytes in plant seeds, termed seed-borne endophytes (SBEs), are of particular interest, as they are vertically transmitted from generation to generation, ensuring their presence in new plants. The property that SBEs can be vertically transmitted through generations makes these endophytes as a kind of plant heritable trait, and the concept of “seed bank”, which was conceived in the 20th century, should include the SBE as an important measure to protect the crop genetic diversity of crops [3,4].

During the past decades, the application of high-throughput sequencing technology has enabled us to rapidly analyze the endophytic communities of plant seeds and other plant tissues, which has greatly expanded our understanding of SBEs. Reviews have focused on the seed endophytes of medicinal plants and woody plants, respectively [5,6]. In this review, we further updated the diversity, origin, systematic, and vertical transmission of SBEs and their effects on host plants, in order to provide references for further research and application of these valuable resources.

## 2. The Taxonomic Diversity of SBEs

SBEs are taxonomically categorized as endophytic bacteria, fungi, and actinomycetes.

### 2.1. Bacterial SBEs

Bacterial SBEs have been reported from a variety of plants including maize, rice, wheat, tobacco, grape, *Eucalyptus*, *Panax ginseng*, Chinese sea buckthorn, and others [6,7,8,9,10], and a large number of bacterial SBEs have been isolated and identified (Table 1). Comprehensively, the majority of the detected bacterial SBEs are from the bacterial phyla Firmicutes and Proteobacteria, and *Bacillus*, *Enterobacter*, *Pseudomonas*, and *Pantoea* are the most commonly detected bacterial genera of plant SBEs. Among these genera, *Bacillus* is a species-rich genus in SBEs. For example, *B. altitudinis*, *B. simplex*, and *B. thuringiensis* were isolated from *Vitis vinifera* L. seeds [10]; *B. subtilis*, *B. megaterium* and *B. proteolyticus* were isolated separately from the seeds of *Helianthus annuus* L. [11], *Eucalyptus* [12], and *Eucommia ulmoides* [13]. *Caragana leucophloea* Pojark seeds contained large amounts of *B. licheniformis*, *B. cereus*, and *B. subtilis* [14]. In addition, a number of bacterial SBEs in *Bacillus* similar genera were identified. *Paenibacillus* spp. are common bacterial SBEs in rice, tobacco, oilseed rape, palms, fescue, and other plants. *Paenibacillus amylolyticus* was identified SBEs in grapevine (*Vitis vinifera* L.) [10]. *Paenibacillus humicus* was detected in *Eucalyptus* seeds [12], and *Eucalyptus* seeds contained diverse bacterial SBEs from the genera *Peribacillus*, *Cytobacillus*, *Metabacillus*, and *Solibacillus* [13], and *Pinus ponderosa* was detected the SBEs from the genus *Psychrobacillus* [6]. In addition, *Sphingomonas*, *Stenotrophomonas*, *Staphylococcus*, and *Enterococcu* were frequently detected bacterial genera in plant seeds. Some common bacteria genera, such as *Serratia*, only detected in maize (*Zea mays* L.), wheat (*Triticum aestivum* L.) and rape (*Brassica napus* L.) seeds [15,16,17], and *Rhizobium* can be isolated from various plants such as honewort (*Bupleurum chinense*), sesbania (*Sesbania cannabina*), rice (*Oryza sativa*) and alfalfa (*Medicago sativa*) seeds [7,18,19] (Table 1).

### 2.2. Fungal SBEs

Hill et al. explored the Millennium seed bank (MSB) as an example of a potential fungal endosymbiotic, using the seed bank of the plant genus *Musa*, where fungi were successfully detected in almost a third of the total seeds, with the majority of OTUs belonging to the fungal genera *Lasiodiplodia*, *Fusarium*, and *Aspergillus*. By assessing the fungal diversity within these stored seeds, it was found that there is a previously overlooked dimension of invisible fungi in the seed bank. And these fungal SBEs have important implications for seed collection and storage, and the collection pathways such as MSB could serve as a new source of useful fungal strains [3].

To date, fungal SBEs can be detected by the traditional isolation methods and by the DNA sequencing-based technologies as well. SBEs in medicinal plants such as ginseng, American ginseng, *Schisandra chinensis*, and *Eucommia ulmoides* have been mostly concerned [18,41,42,43]. Ascomycota and Basidiomycota are dominant plant fungal SBEs, and the main genera of fungal SBEs include *Fusarium*, *Altermaria*, *Penicillium*, *Cladosporium*, and *Saccharomyces* (Table 2). Appropriate 350 fungal SBE strains were isolated from rare bamboo (*Phyllostachys heterocycla* cv. *pubescens*), with the relative abundances of the fungal phyla Ascomycota and Basidiomycetes being 98.0% and 2.0%, respectively [31]. Fungal SBEs in rare bamboos can be categorized into nine orders and nineteen genera, of which four genera *Leptosphaerulina*, *Simplicillium*, *Sebacina*, and an unknown genus in Basidiomycota were newly recorded bamboo endophytic fungi [44]. In *Morinda citrifolia* seeds, 48 fungal SBE strains were isolated, and *Eremothecium coryli*, *Pseudozyma aphidis*, *Cryptococcus flavescens*, *Phaeoacremonium* sp. and *Gibberella* sp. were the dominant SBEs species [31]. The fungal SBEs of the medicinal plant *Corchorus olitorius* was first reported by Ahmed et al. [45], in which 17 strains were identified and categorized into three genera *Penicillium*, *Fusarium*, and *Aspergillus* (Table 2). All these results proved that seeds are repositories for the diversity of fungal SBEs.

### 2.3. Actinomycetic SBEs

Due to their ability to produce multiple antibiotics and other secondary metabolites, actinomycetes have been of great interest as endophytic microbes and can be used as biological pesticides in agriculture [55]. However, few studies have been carried out on the plant actinomycetic SBEs because of the limitations of isolation techniques [56]. Yao et al. isolated 22 strains of seed-borne actinomycetes from *Camptotheca acuminata* Decne, and 21 strains were identified as belonging to the genus *Streptomyces*, and 1 strain belonging to the genus *Nocardiopsis*, by comparing their 16S rDNA in NCBI (national center for biotechnology information) [57]. A large number of strains of actinomycetic endophytes were isolated from *Eucalyptus* seeds and seedlings, among which *Frankiaceae* existed only in seeds [12]. One actinomycetic SBE species, *Streptomyces rochei*, was isolated from *Achnatherum inebrians* [58]. Thirty-two strains of actinomycetic SBEs which belonging to seven genera were isolated from *Sophora alopecuroides* L., and *Streptomyces* was the dominant genus with a relative abundance of 22.5%. The actinomycetic genera *Actinophytocola*, *Saccharothrix*, *Actinosynnema*, and *Glycomyces* were detected only in seeds [59]. In addition, *Micrococcus* was the actinomycetic SBE in *Arabica coffee* [20], and the genus *Microbacterium* was identified in actinomycetic SBE in *Coptis chinensis* Franch. [27]. *Curtobacterium*, as an actinomycetic SBE genus, was detected in a wide range of plants, such as *Bupleurum chinense*, *Oryza sativa*, *Triticum aestivum*, *Areca triandra*, and others [11,17,18,37]. In addition, actinomycetic genera *Micrococcus* and *Frigoribacterium* were isolated from maize seeds [60], and *Micrococcus luteus*, the model species of *Micrococcus*, was isolated and identified from rice seeds [61], and 39 fast-growing thermophilic SBE strains were isolated from CT6919 rice, among which *Curtobacterium* and *Microbacterium* belong to Actinobacteria [62].

## 3. Factors Affect Plant SBEs

The colonization of SBEs by specific plants appeared to be driven by both the genotype of the plant and environmental cues that acted as filters during the process of SBE microbiota establishment.

### 3.1. Plant Genotype and Its SBEs

Similar to other plant endophytes, SBEs have obvious host selectivity. The seed endophytic communities of seven Palmae family plants, including *Areca triandra*, *Caryota mitis*, *Phoenix roebelenii*, *Arenga engleri*, *Livistona chinensis*, *Trachycarpus fortunei*, and *Phoenix canariensis*, were investigated and revealed the obvious genus specificity of the SBE microbiota, only 23 SBEs OTUs shared common among the different tested plant seeds [38]. A cluster analysis showed the great differences in SBEs communities among genotype different cultivars of *Oryza sativa* [63]. After investigating the SBEs communities of four tobacco cultivars separately from Brazil, China, and the United States revealed that cultivars from the same breeding line shared more counts of common OTUs than cultivars from different breeding line, which implied a portion of core SBEs of *Nicotiana tabacum* L. have not been affected during the breeding processes and proportional SBEs have co-segregated with plant genotypes [64]. All these results confirmed the considerable effects of host genotype on SBEs.

The assembly of seed endophytic microbiomes is fundamentally shaped by the dual selection pressures of host metabolic profiles and seed morphology. Extensive studies have demonstrated that intraspecific variation in plant secondary metabolites directly modulates endophyte composition. For instance, certain plant genes, such as the receptor-like kinase shr5 or members of the ethylene signaling pathway, could regulate the plants’ interaction with bacteria and determine whether the bacteria are able to endophytically colonize the plant [65]. This chemotactic selection is further corroborated by Hardoim et al., whose meta-analysis identified consistent correlations between plant-specialized metabolites (e.g., phenolic glycosides, terpenoids) and the enrichment of specific microbial plants [2]. Concurrently, seed morphology imposes physical constraints on microbial colonization. Culturable bacterial populations gradually increased during rice seed development and maturation, and the number of endophytic bacteria gradually decreased with different fates at different storage temperatures [66].

### 3.2. Effects of Environmental Factors on SBEs

In recent years, a number of researchers have detected or isolated endophytes from the same plant seeds in different habitats and found that there are significant differences in SBEs from different habitats. The community structure and diversity of endophytic bacteria and fungi in *Bupleurum chinense* DC. seeds from four provinces were analyzed, and results showed that there were differences at the genus level among seeds with the highest richness and diversity in the seeds of Hebei while the lowest in the seeds of Inner Mongolia, and the predominant strains were similar across regions, but their abundances varied [18]. The seeds of *Hippophae rhamnoides* subsp. *sinensis* from Yuzhong County and Qin’an County (Gansu province, China) were examined, and it was found that the diversity and richness of bacterial SBEs were higher in Yuzhong County, with the dominant genus in Yuzhong County being *Cyanobacteria* and the dominant genus in Qin’an County being *Stenotrophomonas* [26]. In *Chenopodium quinoa* seeds from Nyingchi City, Lhasa City, and Shigatse City (Xizang province, China), 947 strains of fungal SBEs belonging to Ascomycota were isolated, and 24 strains were common to all three regions, with 15 unique species in Lhasa, 10 in Nyingchi, and 9 in Shigatse [47]. In addition, the genus was unique to each region, *Epicoccum* in Nyingchi, *Peyronellaea* in Lhasa, and *Didymella* in Shigatse, and the highest richness, evenness, and diversity of the fungal SBE community was in Shigatse seeds, with no significant differences in species diversity between Nyingchi and Lhasa [47]. The endophytic fungal community showed significant differences in *Elymus nutans* seeds from four different regions of the Tibetan Plateau [67]. In another study, 272 bacterial SBE strains were isolated from the seeds of *Achnatherum splendens* in six different provinces of China, with 41 dominant bacteria belonging to 3 phylum and 14 genera [68]. Overall, SBEs in the same host species from different habitats show different diversity, dominant or unique species.

The observed differences in SBEs among different habitats are likely to be filtered by variations in environmental conditions, such as soil, temperature, and humidity specific to each habitat. The composition of rhizosphere soil microorganisms and fungal SBEs in *Paeonia szechuanica* from four different provenance sites were investigated by Illumina high-throughput sequencing, and the results showed a close association between rhizosphere bacteria and bacterial SBEs, with no significant impact from fungi [36]. Furthermore, soil physical and chemical properties could influence the community structure and abundance of fungal SBEs. Specifically, the diversity of fungal SBEs was significantly negatively correlated with the organic carbon and total phosphorus content, respectively, while the diversity of bacterial SBEs was significantly positively correlated with the total phosphorus content [36]. Apart from the organic carbon and total phosphorus content, SBEs were also influenced by soil pH, although the relationship was not significant. These observations suggested that the structure of microbial community in seeds is closely related to the basic nutrient sources or soil fertility [36]. In another study, the structure of soil microbial communities had a significant impact on the structure of seed microbial communities of the next generation, and the microbial contributions of different soil treatments to seed root systems increased by 90% in the second generation and seed transmission rates improved by 36.3% [69]. Wang et al. found that the survival capability of fungal SBEs of *Ambrosia artemisiifolia* varied significantly under different conditions during overwintering. Under indoor dry conditions, the isolation rate of fungal SBEs decreased with prolonged storage time, indicating that dry indoor environments were unfavorable for the survival of fungal SBEs [70]. The low temperature and precipitation favored the quantity and diversity of fungal SBEs in *Vitis amurensis* Rupr, and the number of fungi obtained from grape tissues in autumn was twice as high as in summer [71].

In addition, environmental stress can also affect the endophytic microbiota of plant seeds. After graphene oxide treatment at 1.2% concentration, the richness and diversity of the fungal SBE communities in ryegrass seeds was significantly increased [72]. Under salt stress conditions, the community profiles in the endophytic communities of salt-sensitive and salt-tolerant rice seeds were greatly changed, the core microbiota in all cultivars of the indica subspecies composed of *Curtobacterium*, *Flavobacterium*, *Enterobacter*, *Xanthomonas*, *Herbaspirillum*, *Microbacterium*, and *Stenotrophomonas* was gradually changed to *Flavobacterium*, *Pantoea*, *Enterobacter*, *Microbacterium*, *Kosakonia*, and *Curtobacterium* [63].

## 4. Acquisition and Transmission of SBEs

**Figure 1:**
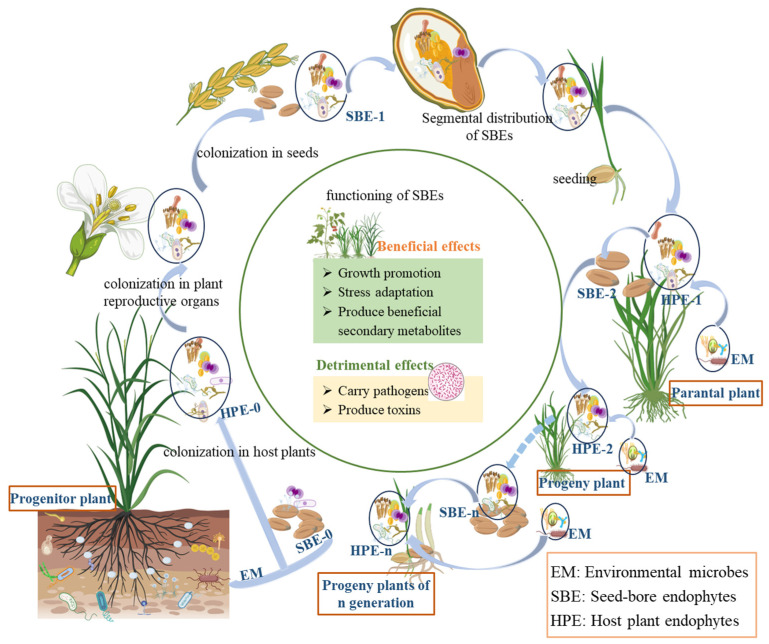
Host effects and cross-generational dynamics of seed-borne endophytes. (Progenitor plant refers to the earliest or ancestral form of a plant species from which other plants have evolved or descended. Parental plant refers to a plant that is directly involved in reproduction, contributing genetic material to offspring through seeds, spores, or vegetative propagation. Solid arrows ‘light blue in the figure’ indicate direct relationships in plant growth, development, and reproduction, while dotted arrows indicate relationships formed through long-term evolution during plant growth and reproduction).

The sources of SBEs are the inheritance of the host’s own endophytes and horizontal acquisition from the external environment, that is vertical transmission and horizontal transmission. Vertical transmission refers to the way that endophytes are transmitted from host to offspring through host seeds or vegetative propagules [65]. Horizontal transmission is the route of transmission by which external microorganisms enter the internal plant through degradation of cellulose in plant cells [73]. SBEs are vertically transmitted from parent to offspring through seeds allowing endophytes to be maintained over generations, thus establishing a close and strong connection with host plants [74,75]. As a result, plants may have evolved an adaptive mechanism to dynamically update their SBE microbiota during reproductive processes through selective inheritance of parental SBEs or acquisition of environmental microorganisms (Figure 1).

### 4.1. Colonization and Transmission of Plant SBEs

Colonization of endophytes into host plants occurs throughout the whole process of plant growth and development. Much research has suggested that most endophytes in plants come from soil microbes. In response to environmental stress, host plants secrete specific chemicals that attract beneficial microorganisms from the surrounding environment, enabling them to resist extreme conditions [76]. The local infection of cucumber roots with *Fusarium oxysporum* has been observed to result in an increase in tryptophan secretion, a decrease in raffinose secretion, and the promotion of the colonization of beneficial bacteria, specifically the *Bacillus amyloliquefaciens* SQR9 strain. This process also serves to reduce the pathogen infestation [8]. In general, once rhizosphere microorganisms are selectively recruited by the host plant, they will show chemotaxis to secretions produced by the root of host plant, migrate, and gather in the rhizosphere of the host plant, then find the opportunity to expand and propagate in the plant, and finally colonize the seeds to form seed endophytes [77]. In addition, root exudates may contain substrates that initiate early communication between host plants and bacterial endophytes, thereby guiding the colonization process. For example, there is evidence that oxalate is involved in the recruitment of the beneficial strain *Burkholderia phytofirmans* by host plants, and oxalate was found to be a material requirement for successful colonization of *Burkholderia* in the rhizosphere after oxalic acid treatment of lupine and maize plants [78]. Studies have shown that soil microorganisms rapidly colonize the root surface in an uneven manner, then further enter the host plant and finally reach the seeds. In this process, the seed coat is first strongly colonized and then the endophyte spreads to the adjacent part [79]. The colonization of grapes by *Burkholderia phytofirmans* strain PsJN under non-sterile conditions was investigated, and results showed that the strain PsJN was detected in a sequential manner on the root surface, in the endodermis and the inflorescence stalk of grapevines [80].

Endophytes gain entry to seeds primarily through three mechanisms:

(1) Transmission from the parent plant to the seed endosperm via vascular tissue. As seeds germinate and develop roots, soil microbiota can enter the plant root system through cracks in the roots or wounds in the plant tissue [81]. Once inside the root inner-layer, bacteria can migrate vertically towards the above-ground parts of the plant through xylem vascular system (with the help of flagella) transpiration streams, or moving along the cellular interstices throughout the mobilization process [82]. Furthermore, certain microorganisms are capable of hydrolysing cellulose in plant cell walls, enabling them to penetrate plant tissues. For example, the wild-type *Burkholderia* strain PsJN secretes large amounts of endoglucanase and endogalacturonase on the surface of grapevine tissues, degrades the plant cell wall, and then enters the internal tissues of the roots, and eventually reaches the seeds [83]. Ferreira et al. studied the mode of endophytes transmission in 10 species of *Eucalyptus* seeds for the first time, and results showed that endophytic bacteria such as *Bacillus*, *Enterococcus*, *Bacteroides*, and *Methylobacterium* could be transferred vertically from seeds to seedlings [12]. The surface of sterilized seeds were inoculated with the endophytic bacteria *Pantoea agloomerans* labeled with the GFP gene. Its presence was detected in root cells and xylem ducts of the stem after seedling formation, proving the vertical transfer of seed microbes through vascular tissue that endophytes could colonize and be transferred to seedlings by seed inoculation [12].

(2) Direct transfer to the endosperm through gametes. When plants enter the flowering and reproduction stages, environmental microorganisms can gain access to the ovary via the stomata on flowers, subsequently colonizing the ovules and seeds [80]. Deckert described in detail the possible ways by which microorganisms or microbial propagators (spores) enter pine seeds: pine trees produce gametes through mitosis, and diploid pollen mother cells undergo meiosis to form four pollen grains. Then, pollen forms pollen tubes in the pollination cavity of the ovule, and sperm enters the ovule through pollen tube germination. Eventually microorganisms enter the ovule and will develop into seeds with the ovule to be seed endophytes if successful colonization [84]. A comprehensive study utilizing maize (*Zea mays*) as a model system systematically analyzed the endophytic communities across different parental lines, pollen, and F1 progeny. The results identified *Bacillus* and *Pantoea* as the predominant endophytic genera in both maize seeds and pollen. Notably, the *Bacillus* diversity profiles in offspring derived from JMC121 and JN728 parental lines exhibited remarkable consistency with those of their respective paternal and maternal sources. Molecular characterization using random amplified polymorphic DNA (RAPD) typing provided compelling evidence for vertical transmission, demonstrating the presence of identical *Bacillus mosieri* strains in paternal line J2416, its pollen, and the resulting JN728 progeny. These findings establish that paternal lines can vertically transmit their dominant endophytic bacteria to offspring through pollen-mediated transfer [85]. Furthermore, studies on rice (*Oryza sativa*) have revealed a conserved “core microbiota” comprising *Herbaspirillum*, *Microbacterium*, *Curtobacterium*, *Stenotrophomonas*, *Xanthomonas*, and *Enterobacter*, which dominate endophytic communities during seed dispersal [86]. This conserved microbial assemblage suggests that parent plants play a crucial role in shaping the endophytic microbiota of their offspring. Alpine grassland species demonstrate similar mechanisms, facilitating the transfer of beneficial endophytes to progeny. This evolutionary adaptation ensures optimal establishment of bacterial symbionts in subsequent generations, with potential applications in crop improvement, microbial restoration, and production of high-quality forage and crop seeds [67].

(3) Transmission through mature fruits. As the host plants grow further to form mature fruits, fruits will be eaten by animals and the seeds will be exposed to soil microorganisms after rumination and defecation. Because of the high microbial diversity of soils, the deposition of seeds into the soil provides extended opportunities for seeds to interact directly with a wide range of soil microbes, so that microorganisms can enter the seeds [87].

### 4.2. Characteristics of Seed-Borne Microorganisms

The transmission of endophytes is the basis for the spread of endophytes through seeds and across generations, which play a pivotal role in host plants. However, not all endophytes are capable of successfully colonizing seeds. In order to successfully colonize seeds, microorganisms must meet the following requirements:

(1) The capacity to synthesize enzymes that facilitate the degradation of plant cell walls, as well as genes responsible for motility and nodulation. Some substances, such as cell wall hydrolase secreted by rhizosphere microorganisms, have the capacity to destroy plant cell walls and thereby circumvent the gating effect of cell walls. The endophytic bacterium *Burkholderia phytofirmans* has the capacity to produce a range of compounds, including cell wall-degrading enzymes such as endoglucanase and cohesive galacturonidase. These enzymes play a role in the degradation of the local cell wall, enabling the bacterium to pass through cracks in the endoderm and subsequently invade and colonize the xylem [88]. Furthermore, microorganisms with flagella and fimbriae exhibit chemotaxis to root secretions produced by host plants when they sense the latter’s presence. They then gather rapidly near the root system of the host plants, forming microbial communities or biofilms at the aggregation site and subsequently adhering to the plant surface [89]. For example, the endophyte Ra36 exhibited flagellate-driven chemotaxis against *Fusarium oxysporum* at alkaline pH, whereas the flagellate-deficient mutant strain did not [90]. Both *Serratia* S119 and *Enterobacter* J49 possess flagella and exhibit swimming, swarming, and twitching motilities. However, they demonstrate disparate chemotaxis to root exudates in the presence of root exudates derived from peanut, maize, and soybean plants [91]. Motility plays a pivotal role in the formation and evolution of biofilms, as well as in their dispersion. Consequently, it represents a crucial mechanism for the colonization and proliferation of endophytes in novel habitats, such as the rhizosphere. Therefore, microorganisms with motility are more likely to colonize seeds than microorganisms without motility [77]. Flavonoids serve as the primary signaling substances for rhizobial colonization. Flavonoids, such as quercetin, isoliquiritigenin, and chickpeaA in peanut root exudates, can significantly contribute to *Phomopsis liquidambaris* mediated peanut-rhizobia nodulation enhancement [92]. Apigenin, a flavonoid present in *Phaseolus vulgaris* and *Lotus japonicus*, has been demonstrated to enhance the expression of nodA1 and nodA2 in *Rhizobia* 899, thereby facilitating their colonization of the root [93].

(2) The ability to adapt to the habitat environment and survive in seeds. Once microorganisms enter the host plant and migrate into the seed to colonize, they need to utilize the seed nutrients available to ensure their survival and reproduction. Most plant endophytes, such as endophytes in rice, generally use the plant’s carbon source to jointly fix nitrogen, which not only promote the plant growth, but also keep the plant in a low nitrogen state, to synthesize and express the nitrogenase of endophytes [94,95].

(3) Quorum sensing, which ensures that microorganisms colonizing the host plant does not interfere with the normal reproduction and growth of the host plant. In rice roots, the PsrR quorum sensing system in the endophyte Cosac (KO348) was involved in improving the colonization [96]. Cinnamoyl homoserine lactone (HSL) signal molecules, produced by the LuxI-LuxR quorum sensing system in *Bradyrhizobium* sp. ORS278, participate in regulating the biofilm synthesis and cell movement, and improving its colonization in rice roots [97].

### 4.3. Distribution of SBEs in Seed Segments

Endophytes are found in the coat, embryo, and endosperm of plant seeds. The diversity of endophytes within the endosperm of wheat seeds was found to be greater than that observed within the embryo. In contrast, the endophytes diversity within the inner rice seed was found to be greater than that observed within the outer shell and the coat [17,98]. Furthermore, the α-Proteobacteria population was more prevalent in the seed-shell and seed-coat than in the seed interior of rice seeds. Conversely, the γ-Proteobacteria population was observed to be more abundant within the interior seed [99]. Therefore, the distribution of endophytes varies among different seed segments.

The composition and abundance of endophytes exhibit significant variations across different developmental stages of the same seed. For instance, the functions of the SBEs in *Amorphophallus muelleri* were driven by their maturation status and that the functions of the microbial communities in the seed coats and seeds were significantly different. During seed maturation, the host exerts a strong selective regulatory effect on the potential functions of plant endophytic microbiome [100]. The endophytic bacteria that were identified at several key stages of dynamic grain growth in maize seeds were studied using the 16S rRNA gene clone library technique. In addition, the diversity and population dynamics of the endophytic bacteria were monitored [15]. The results demonstrated that the species richness of endophytic bacteria exhibited differential patterns across the various stages of seed germination. Notably, the pro-embryonic stage exhibited the highest abundance of endophytic bacteria, surpassing the levels observed in the other two stages [15]. In their study of the changes in microbial diversity during the germination and budding of seeds, Barret et al. observed a significant decrease in the abundance of endophytic fungi and endophytic bacteria in seeds [101].

## 5. Host Effects of SBEs

It was well recognized that the vertical transmission of seed endophytes is a strategy used by plants to cope with environmental challenges. However, there is no report on whether SBEs are involved in the process of seed development. Seeds seem to only serve as carriers for SBEs, which need to transfer these endophytes to the corresponding parts of progeny plants through vertical transmission to exert their effects. The impact of SBEs on host plants may be either beneficial or detrimental (Figure 1).

### 5.1. Beneficial Effects

#### 5.1.1. Growth Promotion

The promotion of plant growth by bacterial SBEs has been extensively studied. Bacterial SBEs are involved in a number of processes that promote plant growth [102], including the production of plant hormones, nitrogen fixation, phosphorus solubilization, secretion of siderophores, and the production of acetyl CoA carboxylase (ACC) deaminase, which have been well summarized and described by Hardoim et al. [2]

Phytohormones are indispensable at every stage of plant development, and some SBEs are capable of synthesizing plant hormones directly or indirectly, thereby promoting plant growth [98]. Plant hormones produced by SBEs include cytokinin (CTK), indole-3-acetic acid (IAA), gibberellic acid (GA), and abscisic acid (ABA) [74]. The bacterial SBEs *Azospirillum lipoferum* from maize and *Bacillus amyloliquefaciens* from rice have the potential to produce gibberellic acid (GA), which interacts with other plant hormones and regulates plant growth at the seedling stage [102,103]. Auxin (IAA) and other metabolites produced by SBEs (*Micrococcus* sp. PB001, *Pseudomonas* sp. PB002, *Methylobacterium* sp. PB005 and Methylorubrum sp. PB009) from rice were enhanced plant growth and grain characteristics of the rice crop [104]. Additionally, some SBEs such as *Burkholderia* sp. and *Rahnella* sp. have been demonstrated to enhance plant growth by facilitating the solubilization of phosphate. And the inoculation of crops with these phosphorus-soluble endophytes resulted in a marked enhancement of growth [105]. Moreover, the fungal SBEs, *Cladosporium* and *Fusarium*, isolated from rice seed in Sri Lanka, could promote rice growth and development by influencing the formation of siderophore [106]. *Bacillus subtilis* HYT-12-1 in tomato seeds with the ability of producing ACC deaminase, which has been shown to inhibit the biosynthesis of ethylene synthesis precursor ACC and relieve the inhibition of ethylene on plant growth, thus significantly promoting the elongation of tomato roots and stems and the accumulation of the biomass [107]. In addition, some endophytic bacteria carry functional genes required for biological nitrogen fixation, such as *Frankia* sp., which enables them to convert nitrogen (N2) into usable forms of nitrogen in the host plant, such as ammonium and nitrate, thereby promoting plant growth [108,109].

In addition to the above, there is a special example of seed endophytes promoting plant growth, orchids. Successful seed germination and seedling growth in orchids require an association with mycorrhizal fungi [110]. Lack of endosperm in their seeds renders orchids to depend on nutrients provided by orchid mycorrhizal fungi (OMF) for seed germination and seedling formation in the wild. OMF that parasitize in germination seeds is an essential element for orchid seedling formation, which can also help orchid reintroduction [111]. Another inoculation experiment also revealed priority effects during root microbiota assembly, where established communities are resilient to invasion by latecomers, and that host preference of commensal bacteria confers a competitive advantage in their cognate host [112]. The reviewed studies provide compelling evidence that SBEs, owing to their distinctive priority colonization advantage in host plants, play pivotal roles in early plant development, including growth promotion, host modulation, and recruitment of other beneficial microbial endophytes.

#### 5.1.2. Stress Adaptation

The close relationship between SBE and seeds gives rise to particular coevolutionary effects that can enhance plant tolerance to a range of stresses, including pathogens, heavy metal, drought and salinity.

Some of the SBEs can enhance the host plant’s resistance to pathogens, such as root rot, leaf spot and leaf blight, caused by *Fusarium oxysporum* [113]. For example, SBEs SLB4-*Pseudomonas fluorescence*, SLB6-*Pseudomonas*, and SY1-*Pseudomonas* could effectively inhibit the infection of *F. oxysporum* on rice seedlings, and SY1 was shown the best inhibition effect, which could promote the growth of rice roots and stems while resisting diseases [114]. The combination of endophytes *Pseudomonas*, *Streptomyces fimicarius*, and *Streptomyces laurentii* could significantly reduce the white leaf wilt of rice, and produce indoleacetic acid, hydrogen cyanide and siderophore to significantly promote plant growth [115]. Endophytes have been shown to enhance the ability of their host plants to cope with pathogen infection by producing a variety of antibacterial substances, including terpenoids, alkaloids, aromatic compounds, lipid polypeptides, ketonolactones, anthrax acid, peptides, and other substances [74,103]. Such as, *Sphingimonas melonis* is capable of producing anthranillic acid, a substance that confers upon it the ability to resist the pathogen *Burkholderia*. Furthermore, this bacterium can be accumulated and transmitted across generations in rice varieties that are resistant to pathogens [28]. *Sphingomonas melonis* that is accumulated and transmitted across generations in disease-resistant rice seeds confers resistance to disease-susceptible phenotypes by producing anthranilic acid because anthranilic acid interferes with the sigma factor RpoS of the seed-borne pathogen *Burkholderia plantarii*, probably leading to the impairment of upstream cascades that are required for virulence factor biosynthesis without affecting cell growth [28]. In addition, SBEs can produce lyases that target chitin, protein, cellulose, hemicellulose, DNA, and other compounds to degrade the cell wall of the pathogen and achieve bacteriostatic purposes. *Withania somnifera* plants treated with endophytic bacteria *Bacillus amyloliquefaciens* and *Pseudomonas fluorescens*, not only increased defense enzymes and antioxidant activity in the treated plants but also enhanced the expression of salicylic acid- and jasmonic acid-responsive genes in the stressed plants, further validating the efficacy of bacterial endophytes against leaf spot disease [116].

At present, the capacity of plants to completely degrade heavy metals is limited; however, certain seed endophytes have been observed to modify the chemical form of metals through endophytic-mediated oxidation or reduction reactions, consequently altering the potential toxicity of metals to plants [117]. There is evidence that endophytic fungi *Epicoccum nigrum* FZT214 in *Dysphania ambrosioides* seeds can increase the content of chlorophyll and glutathione in host plants at different developmental stages, regulating the tolerance of host plants to cadmium stress [118]. Furthermore, the presence of endophytes in *Elymus dahuricus* seeds enhances their resistance to the heavy metal cadmium by modulating the levels and activities of antioxidant enzymes including ascorbate peroxidase, catalase, and superoxide dismutase, proline, and malondialdehyde [119].

Drought and other stresses have been demonstrated to have an impact on the growth process of plants and the expression of genes associated with resistance. Research has indicated that endophytes can enhance the tolerance of host plants to drought stress by regulating the expression of root growth, plant hormones, metabolic processes, and drought resistance genes [120,121]. Endophytes affect the physiological and biochemical status of plants to improve plant tolerance by regulating the expression of genes related to plant cell penetration, metabolism, and photosynthesis. Furthermore, endophytic bacteria can also promote plant resistance by promoting the production and accumulation of active compounds, enhancing plant photosynthesis, and affecting the antioxidant system of the host plant. To date, studies have shown that the production and accumulation of plant active compounds is closely related to endophytes [122].

Apart from that, endophytes can effectively improve metabolic disorders caused by high salt stress in host plants and promote plant growth. Endophytes *Pantoea* and *Bacillus* isolated from seeds of three grass families and four legumes can increase the germination rate of *Medicago sativa* seeds under salt stress [123], and members of *Bacillus* and *Pantoea* in rice seeds can also produce IAA, antagonize fungi, and even have osmotic tolerance [124].

#### 5.1.3. Produce Beneficial Secondary Metabolites

Due to the symbiotic relationship between endophytes and plants, endophytes can produce a variety of beneficial secondary metabolites under suitable environmental conditions and host genotype [125]. Nowadays, with the increasing spread of infectious diseases caused by microorganisms such as bacteria, viruses, and fungi, endophytes have become an important source of pharmacologically active metabolites. For example, the dominant endophytic bacteria *Saccharopolyspora* and *Gordonia* in *Phoenix canariensis* seeds are capable of producing various active secondary metabolites [38]. The endophytic fungus *Psathyrella candolleana* isolated from the seeds of *Ginkgo biloba* can produce various of flavonoids, among which quercetin has strong antioxidant activity, and quercetin, benzoic acid and nicotinamide have antibacterial activity against *Staphylococcus aureus* [126]. *Corchorus olitorius* L. seeds contain abundant endophytic fungal communities with antimicrobial properties: for example, a potent antibacterial substance was extracted from the endophytic fungus *Aspergillus* sp. Ar6, which could be used as source of a new antibacterial chemical for the manufacture of medicines [45]. The study showed that three seed endophytes *Aspergillus* sp. SA1, *Aspergillus* sp. SA2, and *Aspergillus* sp. SA3 isolated from *Nigella sativa* seeds, have inhibitory effect on *Staphylococcus aureus*, *Escherichia coli*, *Pseudomonas aeruginosa*, *Klebsiella pneumoniae*, *Methicillin-resistant staphylococcus aureus* (MRSA) and human pathogen *Candida albicans* because of its various bioactive secondary metabolites, such as polyketides, benzenoids, quinones, alcohols, phenols, or alkaloids [127]. *Pseudozyma aphidis*, the dominant species in *Morinda citrifolia* seeds, is an important strain for lipase production, which can degrade lipids and produce mannose erythritol lipid to differentiate tumor cells and human myeloid leukemia cells, and coordination ability with human immunoglobulin G, which has broad application prospects in the medical field [31].

In addition to their use in the pharmaceutical industry, fungal SBEs can also produce stable pigments and beneficial agents for the development of the food industry. For example, TWSBEF-9, an endophytic fungus isolated from *Tartary buckwheat* seeds, produces red pigment, which is an extracellular pigment and soluble in polar solvents such as water and ethanol [125]. Furthermore, the presence of endophytic fungi *Alternaria*, *Botryosphaeria*, and *Didymella* in *Tartary buckwheat* seeds are significantly correlated with the flavonoid content, suggesting a potential for enhancing flavonoid accumulation and improving buckwheat seed quality [128]. Results also showed that the inoculation of endophytic fungi could increase the activity of superoxide dismutase (SOD), phenylalnine ammonia lyase (PAL), and the content of total phenols (TPh), total flavones (TF) [129,130].

### 5.2. Detrimental Effects

#### 5.2.1. Pathogenicity of Some SBEs

Given that some endophytes are pathogenic, the vertical transmission of seed endophytes may concurrently facilitate the dissemination of certain plant pathogens. Due to their portability, seeds have become a crucial medium for long-distance transmission of pathogens, and a vital conduit for pathogen survival and dissemination [43]. The presence of pathogenic fungi in several Poaceae plant seeds, which were either native to China or introduced from the USA or Canada, was determined. The results demonstrated that the percentage of seed infection in the introduced species was significantly higher than that in the native species [131]. Fungal SBEs was detected from the main producing areas of American ginseng in China, including Liuba in Shaanxi, Fusong in Jilin, Rongcheng in Shandong, and Wendeng in Shandong. It was found that 11 fungal species were identified as pathogens, and *Fusarium* spp., which is considered to be a common soil-borne disease pathogen, was detected in all seed samples [43].

#### 5.2.2. Produce Toxins

The synthesis of harmful toxins by certain seed-borne endophytic fungi has emerged as a key issue requiring urgent attention in agricultural and animal husbandry contexts, as it can affect the quality of host plants and cause poisoning in humans and animals. Based on the previous studies, the alkaloid lolitrem B produced by *Neotyphodium lolii* and Lolium perenne is the major cause of sheep staggers in New Zealand, and ergot alkaloids derived from *Neotyphodium coenophialum* and *Festuca arundinacea* induce ergotism in cattle in the United States, resulting in combined annual economic losses over USD 640 million in the livestock industries of these two countries [132]. Notably, a total of 25 species of grass seeds have been found to harbor *Neotyphodium lolii* in natural grasslands of China, with infection rates reaching 80% to 100%. And *Achnatherum inebrians*, a perennial grass of the Poaceae family that is widespread in the northwestern regions, has long been recognized for its toxicity to domestic animals such as horses, cattle, and sheep. Ingestion of this grass results in symptoms such as mental dullness, reduced feed intake, and uncoordinated gait resembling drunkenness, which are attributed to the presence of endophytic fungi in its seeds [133]. In addition, fusarium head blight (FHB), a global fungal disease caused by various *Fusarium* spp. were transmitted by wheat seeds. The pathogenic *Fusarium* in infected wheat ear produces mycotoxins like deoxynivalenol (DON), 15-acetyldeoxynivalenol (15Ac-DON), and zearalenone (ZEN), and the foodstuffs such as flour produced from them can cause a series of toxic reactions in humans and animals, leading to disorders of the central nervous system and miscarriages in humans and animals in serious cases [134].

## 6. Conclusions and Prospect

SBEs are widely diverse, and increasing research has shown that SBEs are founders of associated microorganisms during the seedling [135], and some that are beneficial play both pro-defensive and defensive roles at different stages of the plant, enhancing the plant resistance by promoting plant growth, improving antioxidant defense, inhibiting the growth of pathogenic bacteria, and producing phytohormones. Seeds, as the crucial reproductive organs of plants, can serve as carriers of endophytes which facilitate mutualistic interactions with plants. Vertically transmitted endophytes (VTEs) with multi-host support functions are considered to be plant-acquired genetic traits that can be regulated to produce plants with stable genetics, which are defined as “plant endophytic modification (PEM)” [136]. Obtaining seeds with targeted genetic traits through the VTE strategy will effectively improve the disadvantage of lengthy cycles in the traditional plant breeding with high economic benefits. However, the endophytes that we have studied are only the tip of the iceberg of all seed endophytes, and a large proportion have yet to be identified or described, limiting our ability to elucidate their functions in plants. In addition, although some beneficial effects of SBEs on the host plants have been discovered, their detail mechanisms and functions need to be further explored. And current research on plant VTEs is still in its infancy, including its scale and mechanism, and the dynamics of SBEs during seed dispersal and transmission are poorly understood, thus it is crucial for understanding the ecological niche of seed endophytes and their long-term survival in natural ecosystems.

The successful application of beneficial seed endophytes requires careful consideration of host-microbe-environment interactions. Key determinants include: (1) host compatibility—select bacterial strains resistant to host-specific antimicrobial metabolites (e.g., benzoxazinoids in cereals) [137,138]; (2) seed architecture—optimize inoculation methods (e.g., vacuum infiltration for thick-coated seeds); and (3) transmission efficiency—prime maternal plants to ensure vertical transfer. Practical strategies should adopt a tiered approach: in vitro screening for metabolic tolerance, in planta validation of colonization patterns using GFP-tagged strains, and field testing under realistic conditions. Emerging tools like microbiome-assisted breeding and synthetic microbial consortia design show promise for enhancing endophyte persistence. Critical gaps remain in understanding how seed dormancy affects microbial survival and whether endophytes can be engineered to bypass host immune recognition. Future research should prioritize developing standardized protocols for seed inoculation and storage, while addressing ecological risks through contained strain engineering. For applied researchers, we recommend: (i) characterizing the native seed microbiome of target cultivars, (ii) matching bacterial functional traits to host needs (e.g., drought tolerance), and (iii) monitoring genomic stability of introduced strains across generations.

Currently, seed coating is considered as one of the best methods to promote sustainable agriculture where the physical and physiological properties of seeds can be improved to facilitate planting. Plant beneficial microorganisms (PBM), such as rhizobia, bacteria, and fungi inoculated via seed inoculation can increase seed germination, plant performance and tolerance across biotic (e.g., pathogens and pests) and abiotic stress (e.g., salt, drought, and heavy metals) while reducing the use of agrochemical inputs [139].

Endophytic microorganisms have emerged as promising candidates for developing novel bioinoculants and biopesticides, presenting several advantages compared to synthetic agrochemicals. Their unique capacity to establish symbiotic relationships with host plants allows for more sustainable and targeted agricultural applications. Unlike chemical alternatives, endophyte-based solutions demonstrate improved environmental safety profiles while maintaining consistent performance throughout the crop cycle [140]. However, we still face many constraints and challenges in developing large-scale applications in sustainable agriculture. In recent years, agriculture has developed rapidly and the use of large amounts of fertilizers has led to varying soil degradation to some degree, threatening plants and rhizosphere microorganisms. Therefore, it is essential to explore beneficial SBEs, study their functions and routes of transmission, and apply in agriculture, biological control, and environmental remediation.

## Figures and Tables

**Table 1:** The frequently detected bacterial SBEs genera (species) in plants.

Host Plant	Detected Bacterial SBEs
*Pinus virginiana* Mill. [6]	*Erwinia*, *Sphingomonas*, *Enterobacter*, *Gammaproteobacteria*, *Pantoea*, *Bacillus*, *Staphylococcus*
*Pinus ponderosa* Douglas ex C. Lawson. [6]	*Lysinibacillus*, *Psychrobacillus*, *Bacillus*, *Pseudomonas*
*Sesbania cannabina* (Retz.) Pers. [7]	*Bacillus*, *Rhizobium*, *Cellulomonas*
*Vitis vinifera* L. [10]	*Bacillus altitudinis*, *Bacillus simplex*, *Bacillus thuringiensis*, *Paenibacillus amylolyticus*, *Staphylococcus aureus*
*Helianthus annuus* L. [11]	*Bacillus subtilis*, *Enterobacter*
*Eucalyptus* spp. [12]	*Bacillus megaterium*, *Enterococcus mundtii*, *Methylobacterium variabile*, *Methylobacterium gregens*, *Paenibacillus humicus*, *Sphingomonas phyllosphaerae*
*Eucommia ulmoides* Oliv. [13]	*Peribacillus*, *Cytobacillus*, *Metabacillus*, *Solibacillus*, *Bacillus proteolyticus*
*Caragana leucophloea* Pojark. [14]	*Sphingomonas*, *Bacillus*, *Bacillus licheniformis*, *Bacillus cereus*, *Bacillus subtilis*
*Zea mays* L. [15]	*Pseudomonas*, *Pantoea*, *Sphingomonas*, *Enterobacter*, *Stenotrophomonas*, *Serratia*
*Brassica napus* L. [16]	*Bacillus*, *Ochrobactrum*, *Alcaligenes*, *Brevundimonas*, *Stenotrophomonas*, *Vitreoscilla*, *Achromobacte*, *Pseudomonas*, *Pseudochrobactrum*, *Sphingomonas*, *Serratia*, *Providencia*
*Triticum aestivum* L. [17]	*Paenibacillus*, *Acidovorax*, *Pantoea*, *Burkholderia*, *Serratia*, *Lactobacillus*, *Massilia*, *Neisseria*, *Methanobacterium*, *Chryseobacterium*
*Bupleurum chinense* DC. [18]	*Pseudomonas*, *Rhizobium*, *Sphingomonas*
*Medicago sativa* spp. [19]	*Rhizobium*
*Coffea arabica* L. [20]	*Burkholderia*, *Stenotrophomonas*, *Bacillus*, *Yersinia frederiksenii*
*Picea abies* (L.) H. Karst. [21]	*Pseudomonas*, *Rahnella*
*Quercus palustris* Münchh [22]	*Pseudomonas*, *Propionibacterium*, *Erwinia*
*Cinchona ledgeriana* (Rubiaceae) Moens exTrim. [23]	*Diaporthe*
*Fraxinus* spp. [24]	*Pantoea*, *Rhodococcus*, *Exiguobacterium*, *Staphylococcus*, *Bacillus*
*Dendrobium nobile* Lindl. [25]	*Pantoea*, *Pseudomonas*, *Acinetobacter*, *Dechloromonas*, *Vibrio*
*Hippophae rhamnoides* subsp. *Sinensis* [26]	*Stenotrophomonas*, *Phyllobacterium*, *Variovorax*, *Cyanobacteria*, *Bacillus*, *Staphylococcus*, *Pseudomonas*, *Acinetobacter*
*Coptis chinensis* Franch. [27]	*Bacillus*, *Stenotrophomonas*, *Achromobacte*,
*Oryza sativa* L. [28,29]	*Bacillus*, *Pseudomonas*, *Paenibacillus*, *Acidovorax*, *Pantoea*, *Sphingomonas*, *Burkholderia*, *Rhizobium*
*Ginkgo biloba* L. [30]	*Bacillus*, *Pseudomonas*
*Morinda citrifolia* L. [31]	*Bacillus*, *Acinetobacter*
*Nicotiana tabacum* L. [32]	*Bacillus*, *Pseudomonas*, *Paenibacillus*, *Pantoea*, *Stenotrophomonas*
*Cicer arietinum* L. [33]	*Bacillus*, *Pseudomonas*, *Staphylococcus*, *Pantoea*, *Enterobacter*
*Solanum lycopersicum* L. [34]	*Bacillus*
*Vicia faba* L. [35]	*Bacillus*, *Bacteroides*, *Lactobacillus*
*Paeonia szechuanica* [36]	*Leptospirillum*, *Lactobacillus*, *Helicobacter*, *Acidiphilium*, *Renibacterium*
*Salvia miltiorrhiza* Bunge [37]	*Pseudomonas*, *Pantoea*, *Sphingomonas*
*Trachycarpus fortune* (Hook.) H. Wendl. [38]	*Enterococcus*, *Paenibacillus*
*Areca triandra* Roxb. exBuch.-Ham. [38]	*Enterococcus*, *Paenibacillus*
*Caryota mitis* Lour. [38]	*Enterococcus*, *Sphingomonas*
*Phoenix roebelenii* O. Brien [38]	*Enterococcus*, *Cellulomonas*, *Methylobacterium*, *Sphingomonas*
*Arenga engleri* Becc. [38]	*Enterococcus*, *Paenibacillus*
*Livistona chinensis* (Jacq.) R. Br. exMart. [38]	*Lactococcus*, *Oceanobacillus*
*Phoenix canariensis* Chabaud [38]	*Saccharopolyspora*, *Kosakonia*, *Enterobacter*, *Goodfellowiella*
*Panax notoginseng* (Burk.) F. H. Chen [39]	*R-proteobacteria*, *Pseudomonas*, *Enterobacter*, *Stenotrophomonas*
*Festuca ovina* L. [40]	*Bacillus*, *Paenibacillus*, *Pseudomonas*

**Table 2:** The frequently detected fungal SBEs genera (species) in plants.

Host Plant	Detected Fungal SBEs
*Eucalyptus* spp. [12]	*Alternaria*
*Eucommia ulmoides* Oliver [13]	*Cunninghamella*, *Aspergillus*, *Penicillium*, *Talaromyces*, *Cladophialophora*, *Alternaria*, *Periconia*, *Cladosporium*, *Arthrinium*, *Trichoderma*, *Bjerkandera*
*Bupleurum chinense* DC. [18]	*Papiliotrema*, *Filobasidium*, *Aspergillus*
*Quercus palustris* Münchh [22]	*Alternaria*, *Debaryomyces*, *Apiognomonia*, *Mycosphaerella*, *Malassezia*, *Aureobasidium*, *Epicoccum*
*Dendrobium nobile* Lindl. [25]	*Alternaria*, *Debaryomyces*, *Ruistroemia*, *Entoloma*, *Psathyrella*
*Morinda citrifolia* L. [31]	*Eremothecium coryli*, *Pseudozyma aphidis*, *Pseudozyma hubeiensis*, *Cryptococcus flavescens*, *Kodamaea ohmeri*, *Cladosporium sphaerospermum*, *Phaeoacremonium*, *Gibberella*, *Penicillium*
*Paeonia szechuanica* Fang [36]	*Penicillium*, *Geomyces*, *Aspergillus*, *Gibberella*, *Rhizopus*, *Lichtheimia*
*Salvia miltiorrhiza* Bunge [37]	*Talaromyces*
*Schisandra chinensis* (Turcz.) Baill. [41]	*Penicillium*, *Penicillium thomii*, *Penicillium expansum*
*Panax ginseng* C. A. Mey. [42]	*Fusarium*, *Cephalotheca*, *Podospora*, *Wardomyces*, *Haematonectria*
*Panax quinquefolius* L. [43]	*Acremonium*, *Fusarium*, *Cladosporium*, *Gibberella*
*Phyllostachys edulis* (Carr.) H. De Lehaie [44]	*Leptosphaerulina*, *Simplicillium*, *Sebacina*
*Corchorus olitorius* L. [45]	*Penicillium*, *Fusarium*, *Aspergillus*
*Phyllostachys heterocycla* cv. *Pubescens* [46]	*Colletotrichum*, *Cladosporium cladosporioides*, *Shiraia bambusicola*
*Chenopodium quinoa* Willd. [47]	*Alternaria*, *Fusarium*, *Phoma*, *Cladosporium*, *Peyronellaea*, *Epicoccum*, *Didymella*
*Acer ginnala* Maxim [48]	*Alternaria*, *Epicoccum*
*Lodgepole pine* Parl. [49]	*Alternaria*
*Quercus petraea* Liebl [50]	*Taphrina carpini*, *Cladosporium delicatulum*, *Epicoccum nigrum*, *Curvibasidium cygneicollum*
*Acer palmatum* Thunb. [51]	*Colletotrichum*, *Septoria*
*Larix gmelinii* (Rupr.) Kuzen. [51]	*Rhodotorula*, *Didymella*, *Cystobasidium*
*Pinus* spp. [51]	*Kabatina*
*Fagus* spp. [51]	*Parastaganospora*
*Gardenia jasminoides* Ellis. [52]	*Aspergillus*, *Penicillium*, *Mucor*, *Rhizopus*
*Torreya yunnanensis* Cheng et L. K. Fu. [53]	*Alternaria*, *Trichoderma*, *Phomopsis*, *Fusarium*, *Colletotrichum*, *Paecilomyces*
*Cinnamomum longepaniculatum* (Gamble) N. Chao [54]	*Pestalotiopsis*, *Parastaganospora*, *Aspergillus*, *Paraconiothyrium*, *Peniophora*, *Cryptodiscus*, *Penicillium*

## Data Availability

The original contributions presented in this study are included in the article. Further inquiries can be directed to the corresponding authors.

## Abbreviations

The following abbreviations are used in this manuscript:

EM	Environmental microbes
SBE	Seed-bore endophytes
HPE	Host plant endophytes
VTE	Vertically transmitted endophytes
PBM	Plant beneficial microorganisms
